# Behavioural responses to mammalian grazing expose insect herbivores to elevated risk of avian predation

**DOI:** 10.1098/rspb.2024.1112

**Published:** 2024-10-09

**Authors:** Yu Zhu, Xiaofei Li, Xiongbing Tu, Anita C. Risch, Zhaojun Wang, Quanhui Ma, Ming Jiang, Yuanchun Zou, Deli Wang, Moshe Inbar, Dror Hawlena, Zhiwei Zhong

**Affiliations:** ^1^ Ministry of Education Key Laboratory of Ecology and Resource Use of the Mongolian Plateau & Inner Mongolia Key Laboratory of Grassland Ecology & Observation and Research Station for the Typical Steppe Ecosystem of the Ministry of Education, School of Ecology and Environment, Inner Mongolia University, Hohhot 010021, People’s Republic of China; ^2^ State Key Laboratory of Black Soils Conservation and Utilization & Heilongjiang Xingkai Lake Wetland Ecosystem National Observation and Research Station & Key Laboratory of Wetland Ecology and Environment, Northeast Institute of Geography and Agroecology, Chinese Academy of Sciences, Changchun 130102, People’s Republic of China; ^3^ College of Resources and Environmental Sciences, Jilin Agricultural University, Changchun 130118, People’s Republic of China; ^4^ State Key Laboratory for Biology of Plant Diseases and Insect Pests, Institute of Plant Protection, Chinese Academy of Agricultural Sciences, Beijing 100193, People’s Republic of China; ^5^ Swiss Federal Institute for Forest, Snow and Landscape Research WSL, Birmensdorf 8903, Switzerland; ^6^ School of Environment, Northeast Normal University, Changchun 130117, People’s Republic of China; ^7^ Institute of Grassland Science, Key Laboratory of Vegetation Ecology, Ministry of Education/Jilin Songnen Grassland Ecosystem National Observation and Research Station, Northeast Normal University, Changchun 130024, People’s Republic of China; ^8^ Department of Evolutionary and Environmental Biology, University of Haifa, Haifa 3498838, Israel; ^9^ Department of Ecology Evolution, and Behavior, The Alexander Silberman Institute of Life Sciences, The Hebrew University of Jerusalem, Jerusalem 91904, Israel; ^10^ Key Laboratory of Grassland Resources (Inner Mongolia Agricultural University), Ministry of Education, Hohhot 010021, People’s Republic of China

**Keywords:** large herbivores, predator–prey interactions, predation risk, birds, grasshoppers, trophic cascades

## Abstract

Large mammalian herbivores (LMH) are important functional components and drivers of biodiversity and ecosystem functioning in grasslands. Yet their role in regulating food-web dynamics and trophic cascades remains poorly understood. In the temperate grasslands of northern China, we explored whether and how grazing domestic cattle (*Bos taurus*) alter the predator–prey interactions between a dominant grasshopper (*Euchorthippus unicolor*) and its avian predator the barn swallow (*Hirundo rustica*). Using two large manipulative field experiments, we found that in the presence of cattle, grasshoppers increased their jumping frequency threefold, swallows increased foraging visits to these fields sixfold, and grasshopper density was reduced by about 50%. By manipulatively controlling the grasshoppers’ ability to jump, we showed that jumping enables grasshoppers to avoid being incidentally consumed or trampled by cattle. However, jumping behaviour increased their consumption rates by swallows 37-fold compared with grasshoppers that were unable to jump. Our findings illustrate how LMH can indirectly alter predator–prey interactions by affecting behaviour of avian predators and herbivorous insects. These non-plant-mediated effects of LMH may influence trophic interactions in other grazing ecosystems and shape community structure and dynamics. We highlight that convoluted multispecies interactions may better explain how LMH control food-web dynamics in grasslands.

## 1. Introduction

Large mammalian herbivores (LMH) are a critically important component of many terrestrial ecosystems [1–3]. Globally, the diversity and abundance of wild LMH have dramatically declined owing to habitat degradation and human exploitation [4–6]. At the same time, LMH—including livestock or alien species—are increasingly being introduced into ecosystems for commercial and conservation purposes, even to habitats that historically lacked large mammalian grazers [7,8]. These rapid changes in LMH assemblages and distribution patterns may perturb food-web dynamics [9,10], cascading to affect key ecosystem structure and functioning, such as primary productivity and elemental cycling [11–13]. A crucial step in predicting such changes in ecosystems is to explore the pathways by which LMH grazing may affect food-web dynamics. Given the importance and widespread nature of LMH, determining how they affect other species, and the direction and magnitude of these interactions, is critical for fully understanding community structuring. Despite the importance of LMH as drivers of community structure and the dynamics of plant and associated fauna, their role has not been well integrated into community and food-web ecology (but see [9,10]). Hence, we know remarkably little about how these mammals structure ecological communities.

LMH may exert profound direct and indirect effects on food-web and ecosystem dynamics [14–16]. In this respect, most research has focused on plant-mediated interactions. LMH control the abundance, distribution and diversity of plants via consumption, nutrient recycling and trampling [8,17,18]. LMH also compact the soil, which makes it harder for plants to grow roots, and they also exacerbate erosion in steep terrains, particularly during rainfall events [19]. Subsequently, these changes cascade to affect other primary and secondary consumers and control food-web dynamics. For instance, LMH remove plant biomass and limit populations of smaller herbivores via resource competition [9,20–22]. Additionally, removal of plant biomass may expose smaller herbivores to harsh physical environments or elevated risks of predation by simplifying the habitat structure [14,23].

Potentially important but less explored are the non-plant-mediated processes through which LMH regulate food-web dynamics [24,25]. Owing to significant asymmetries in body size, LMH can directly affect smaller, plant-dwelling invertebrate herbivores through unintentional ingestion or trampling [14,25,26]. When facing these imminent risks from LMH, small invertebrates—both herbivores and predators—often respond defensively by jumping-off or flying out of the vegetation canopy [27–30]. These defence mechanisms may, in turn, expose small invertebrates to higher predation risks from insectivorous predators, such as birds [31–33]. Possibly the best-known example of such an interaction is the increase in the hunting efficiency of cattle egrets (*Bubulcus ibis*) that forage beside cattle (see [34,35] and references therein). As cattle move through the fields, they disturb insects hidden within the vegetation, thereby rendering them more visible and accessible to cattle egrets. Therefore, LMH have the potential to alter trophic interactions by modifying the behaviours of co-occurring animal species and *per capita* interaction strength between preys and predators. Yet despite being long-acknowledged, such convoluted multi-species interactions have received little attention, leaving their mechanistic details and ecological importance largely unknown.

**Figure 1.**
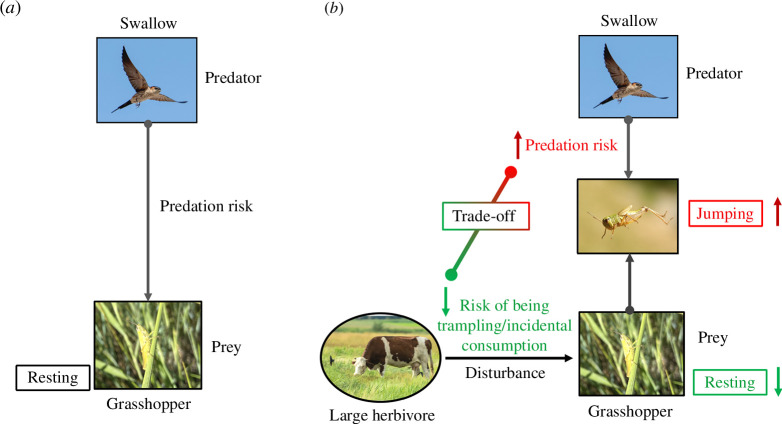
The hypothesized interactions among avian predators (*H. rustica*), insect prey (*E. unicolor*) and the effects of large mammalian herbivores (cattle, *B. tarurs*) in an east-Asian steppe community. In the absence of herbivore grazing, swallows should rarely consume undisturbed grasshoppers resting on vegetation canopy (figure 1*a*). In the presence of herbivore grazing, however, cattle movements may cause grasshoppers to jump out of the vegetation cover to escape incidental predation or trampling (indicate as green box and arrows), exposing them to risk of swallow predation (indicate as red box and arrows), resulting in a trade-off between the two types of risks (figure 1*b*).

To bridge this knowledge gap, we explored the three trophic interactions between cattle (*Bos taurus*), grasshopper (*Euchorthippus unicolor*) prey, and barn swallow (*Hirundo rustica*) predators in a temperate grassland in northeast China. Grasshoppers are prevalent herbivorous insects in grasslands and they play an important role in regulating the structure and function of these ecosystems [36,37]. Grasshoppers tend to forage within the vegetation canopy, which provides a refuge from avian predation. Barn swallows are a common avian predator in this ecosystem that hunt for flying insects almost exclusively on the wing [38]. We tested the hypothesis that grasshoppers, by jumping out of vegetation cover to avoid the approaching grazing cattle, are prone to higher risk of swallow predation (figure 1).

We first conducted a grazing experiment in the field to test the effects of cattle grazing on the jumping behaviour and abundance of grasshoppers and on the visiting rate of their swallow predators. We then manipulated the grasshoppers’ ability to jump and tested whether the change in their jumping behaviour (jumping constrained) increased the grasshoppers' probability of being killed by cattle, and whether it reduced their vulnerability to swallow predation. Since grasshopper males are more active than females, we also tested whether their vulnerability to swallow predation is sex-dependent [39]. Our work highlights the importance of LMH grazing in regulating food-web dynamics via pathways that are not mediated by plants.

## 2. Methods

### (a) Study site and background

The study was conducted in a semi-arid grassland in Changling county, Jilin Province of northeast China (45°14′ N, 123°21′ E). The study site has a semi-arid continental monsoon climate. Annual mean temperature is 4.3°C (varying from −18°C in January to 23°C in July) and average annual precipitation is 413 mm, with over 70% falling during the growing season from June to August [40]. The plant community in this grassland is predominated by the perennial grass *Leymus chinensis*. Other common species include the grasses *Phragmites australis*, *Chloris virgata* and *Setaria viridis*, and the forbs *Artemisia scoparia*, *Ixeris denticulata* and *Polygonum sibiricum*. Historically, cattle and horses grazed these grasslands, but starting in the 1990s, the study area was fenced to exclude livestock and human residents for grassland management and conservation purposes [41]. While low densities of rabbits and voles may occur in this grassland, these species are primarily browsers.

*Euchorthippus unicolor* (female: 20–23 mm, male: 14–17 mm) is an abundant grasshopper species in this ecosystem, accounting for >50% of all insect abundance [41]. This grasshopper emerges in early July and reaches a peak density of 5–10 individuals per m^2^ in August [42]. *Euchorthippus unicolor* primarily feeds on the dominant *L. chinensis* grass, and only infrequently on forbs. The major predators of grasshoppers in this ecosystem include spiders and various birds [10]. The barn swallow (*H. rustica*) is an important avian predator in this grassland that hunts mainly by flying above open grasslands or shallow water in search of flying insects like flies, wasps, aphids and beetles. Barn swallows also follow large mammals, humans and farm machinery to catch disturbed insects like crickets and grasshoppers that jump out of the vegetation canopy (Z. Z., personal observation; see electronic supplementary material, figure S1).

### (b) Grazing experiment

An experiment was conducted in 2020–2022 to assess the effects of cattle grazing on the behaviour and the abundance of swallows and grasshoppers and their trophic interaction. We randomly positioned six blocks of two 50 × 50 m plots within the study area (electronic supplementary material, figure S2). The six blocks were separated from each other by 150–300 m. Within each block the distance between the two plots was approximately 30 m (electronic supplementary material, figure S2). One plot in each block was randomly assigned to cattle grazing and the other was used as an ungrazed control. The grazed plots experienced cattle grazing of light to moderate intensity (0.1–0.3 animal units ha^−1^), as recommended by the local government. Grazing occurred each year from June to August during the first two weeks of each month, with daily grazing occurring between 08.00−11.00 and 13.00−16.00, simulating local practice.

To understand the effects of cattle grazing on interactions between swallows and grasshoppers, we quantified the behaviours and abundance of both grasshoppers and swallows in the twelve 50 × 50 m ungrazed and grazed plots during the summer of 2021. Our observations were performed twice, in mid- and late-August, with each survey carried out for 1 day between 08.00−11.00 and 13.00−16.00, corresponding to the peak activity periods for grasshoppers, swallows and cattle. The analyses of the behaviours of swallows and grasshoppers was based on 144 h of observation for each measure (12 plots × 6 h day^−1^ × 2 days). For cattle behaviour, we had a total of 72 h of observation (6 plots × 6 h day^−1^ × 2 days).

To evaluate swallow behaviour, we visually assessed the number of swallow visits in each plot. A ‘visit’ was defined as a flying swallow diving down to less than 2 m above the vegetation. For grasshopper behaviour, we randomly established two 4 × 4 m permanent quadrats separated by 10 m at the centre of each plot (see electronic supplementary material, figure S2). These quadrats allowed cattle (in the grazed plots) to walk freely and disturb the grasshoppers. We visually quantified the total number of jumping grasshoppers in these quadrats. For cattle behaviour, we randomly selected two individuals and counted the total number of steps they took (only in the grazed plots). A ‘step’ was defined as a single step with one of the front legs. To minimize disturbances by the observer, we ensured that all observers wore similar camouflage clothing and sat beside the two permanent quadrats (for grasshopper behaviour) and along the fences (for swallow and cattle behaviour) of each plot [43]. Observations were made by the human eye and, when needed, with the aid of a telescope (SY-40x22, SXVETWP Optical Instrument, Zhejiang, China). We first pooled the grasshopper data from the two permanent quadrats and the step data from two cattle for each sampling date, respectively. We then averaged all behavioural data of swallows, grasshoppers and cattle from the two sampling dates and used these data for the statistical analyses.

In mid- and late-August 2021, we also determined the effects of cattle grazing on grasshopper density in all plots (electronic supplementary material, figure S2). In each plot, we set two parallel 50 m transects with 20 m distance between them. On each transect we placed five 0.1 m^2^ rings in 8 m intervals. These rings remained undisturbed for 2 days before the surveys. We counted the number of *E. unicolor* grasshoppers within each ring to estimate their density by slowly approaching each ring and counting the jumping grasshoppers and those that remained within the vegetation [44]. All surveys were conducted on sunny days with minimal cloud cover and no wind. We first averaged grasshopper density for the two transects and then for the two sampling dates to obtain a single value for each plot for statistical analyses.

Finally, we measured vegetation community composition in mid-August 2021 to determine the effects of grazing on plant community properties in the plots. Vegetation was assessed in five 1 × 1 m quadrats along each transect that was used to investigate grasshopper density (electronic supplementary material, figure S2). Within each quadrat, vegetation was divided into three functional groups: *L. chinensis* grass (host food plants for grasshoppers), other grasses and forbs, and their percentage cover was visually estimated [41]. We averaged plant cover of each functional group for the two transects (a total of 10 quadrats) to obtain a single value for each plot for statistical analyses.

### (c) Grasshopper behaviour manipulation experiment

In August 2022, we investigated how the jumping behaviour of grasshoppers can alter their mortality risks from incidental consumption/trampling by cattle and from swallow predation using three experimental treatments: (i) grasshoppers that were able to jump in an ungrazed plot, (ii) grasshoppers that were able to jump in grazed plots, and (iii) grasshoppers that were unable to jump in grazed plots. We only manipulated grasshopper jumping ability in the grazed plots to specifically examine whether and how such shifts in prey behaviour can modify trophic interactions among cattle, grasshoppers and avian predators. The first treatment was used as a 'no grazing' reference.

*Euchorthippus unicolor* grasshoppers were collected from a natural site near the experimental grazing blocks that experienced no mowing or grazing. Within each of the ungrazed and grazed plots, we randomly tethered 50 male and 50 female adult grasshoppers to leaves of their main food plant, the *L. chinensis* grass. We used a 1 m piece of 1 mm diameter monofilament line to tether each grasshopper. The filament 1 m length corresponds to the maximum jumping distance of *E. unicolor*, allowing the tethered grasshoppers to jump freely (Y. Z., 2019, field observations). This length also reduced the risk that grasshoppers would break free by chewing the line [39]. In the grazed plots, we assigned an additional treatment to limit the jumping ability of 100 grasshoppers (50 male and 50 female) by tethering them to the vegetation with only 0.1 m long monofilament line. This distance corresponds to the typical length of the grasshoppers’ favoured *L. chinensis* leaves. The 0.1 m lines allowed the grasshoppers to forage within the vegetation, but prevented them from jumping out of the vegetation canopy. All tethered grasshoppers were randomly placed at a distances of more than 2 m from each other to avoid potential interference. This experiment was repeated three times on 1st, 7th and 13th August, 2022. A total of 5400 individual grasshoppers were used in the behaviour manipulation experiment (100 individuals × 3 treatment × 6 replicates ×3 times).

The grasshoppers were tethered in the field before the onset of cattle grazing and remained exposed to swallow predation between 08.00 and 16.00. At this time, we counted the numbers of male and female grasshoppers killed by two sources of risk: incidental consumption/trampling of cattle and by predatory swallows. We determined the source of the grasshoppers’ mortality by examining characteristic signs on the tethered grasshoppers. Cattle often chew once or twice and then immediately spit out the entire grasshoppers from their mouths (Y. Z., 2019, personal observation). Hence, incidental consumption or trampling by cattle resulted in crushed but otherwise complete grasshopper carcasses (electronic supplementary material, figure S1). Tethered grasshoppers were susceptible to various predators, including swallows, spiders and ants. When swallows attacked grasshoppers, they typically flew away with the abdomen owing to the difficulty in breaking the tether, leaving the head and legs of the grasshopper behind. Consequently, the grasshopper carcasses were often incomplete (electronic supplementary material, figure S1). We deleted from the analyses grasshoppers that were consumed by spiders or ants. We determined spider predation when directly observing a spider feeding on a grasshopper or when it was found in a web [39]. Ants typically scavenge on grasshoppers that are [45]. However, owing to the difficulty in determining the cause of death of these dismembered grasshoppers, we did not include these data in the analyses. At the end of each experiment, we recorded whether the tethering line was broken. We replaced each missing grasshopper with a new individual of the same sex.

We used the formula: (the number of male/female grasshoppers killed by cattle (or by swallows)/50) × 100% to obtain the mortality risk of male and female grasshoppers from incidental consumption/trampling by cattle and by predation from swallows in the three experimental treatments above.

### (d) Statistical analyses

All data were analysed using the open-source software R v. 4.3.0 [46]. All models were tested for normality of residuals using the Shapiro–Wilks test and the visual inspection of residuals versus fitted values [47].

The effects of cattle grazing on grasshoppers and swallows in 2021 were first analysed using linear mixed effect models. We used grazing treatment (ungrazed or grazed) as a fixed effect and block as a random effect to assess the effects of cattle grazing on swallow visiting frequency, the number of jumping grasshoppers and grasshopper density in the plots. The difference in vegetation community in grazed and ungrazed plots was tested using the same model. The response variable assessed was plant cover for each functional group (*L. chinensis*, other grasses and forbs). Models were fitted using the function lmer from the package lme4 [48] and the package lmerTest was used to calculate *p*-values [49]. We then tested for relationships among cattle walking steps, the number of jumping grasshoppers and swallow visiting frequency in the grazed and ungrazed plots using a linear regression.

To assess the effects of grasshoppers’ jumping behaviour on the mortality risk from cattle and predatory swallows (grasshopper behaviour manipulation experiment) in 2022, two separate analyses were conducted. First, we omitted the extra ‘grazed–no jumping’ treatment and analysed data from the remaining two treatments (‘ungrazed’ and ‘grazed–jumping’) using a generalized linear mixed model with a binomial error structure, in which grazing treatment and grasshopper sex were treated as factorial fixed factors and sampling time (the behaviour experiment was repeated three times) as a random factor. This allowed us to test for the main effects of cattle grazing and sex of grasshoppers and their interaction on the grasshoppers' probability of being killed by swallows. The response variable in the model was a two-column dataset recoding the number of dead and alive grasshoppers. A second analysis was used to assess the differences in the proportions of male and female grasshoppers killed by cattle or swallows among the three treatments (‘ungrazed’, ‘grazed–jumping’, ‘grazed–no jumping’) to test whether shifts in jumping behaviours were the key determinant of grasshopper mortality from cattle and swallows. If a significant effect was detected, we conducted Tukey multiple comparison analyses from the package emmeans [50] to assess the differences among specific treatments.

## 3. Results

### (a) Grazing experiment

**Figure 2.**
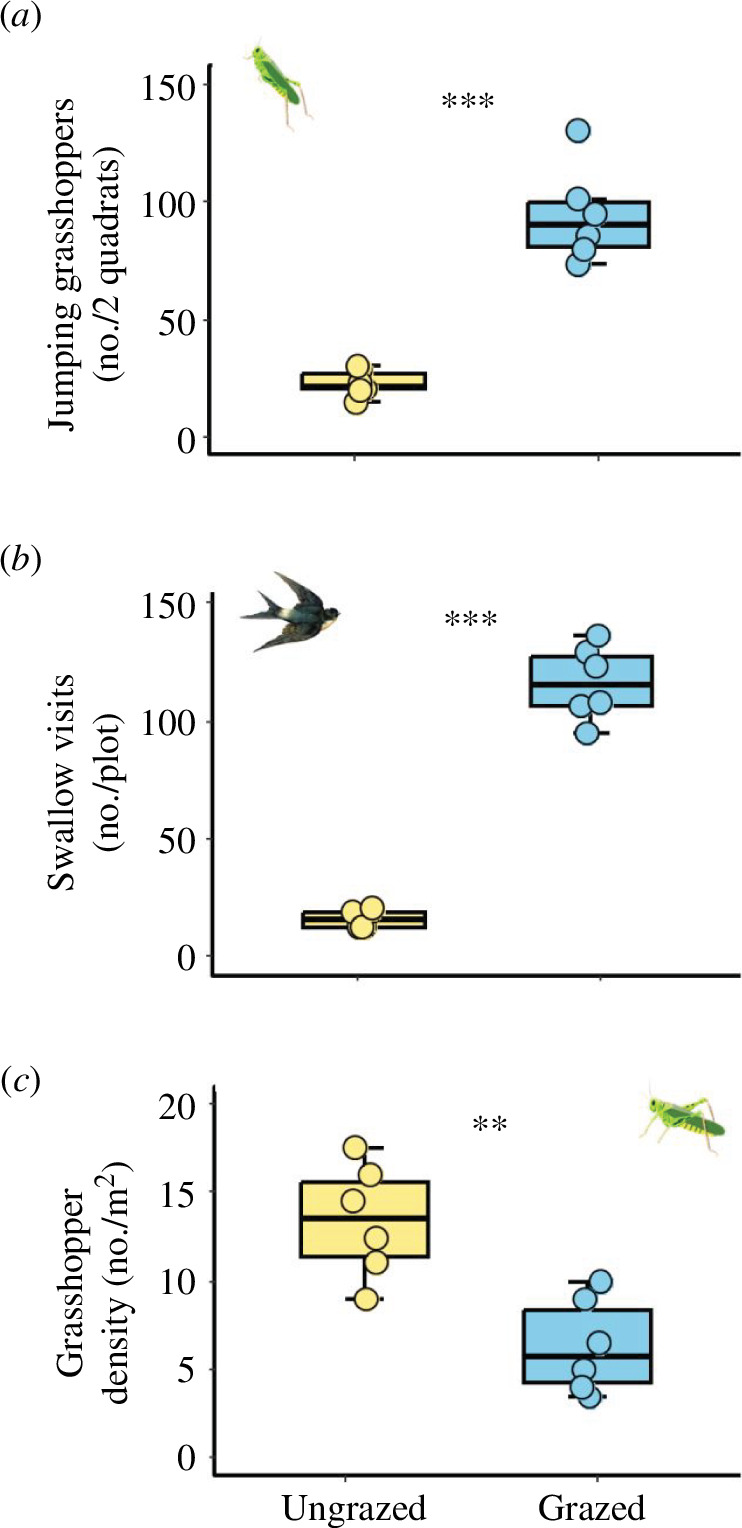
Effects of cattle grazing on (*a*) the number of jumping grasshoppers, (*b*) swallow visit frequency and (*c*) grasshopper abundance in the six ungrazed and grazed plots in August of 2021. Asterisks between the bars indicate significant differences between grazing treatments (**p ≤ 0.01, ***p ≤ 0.001). Error bars represent ± s.e.

**Figure 3.**
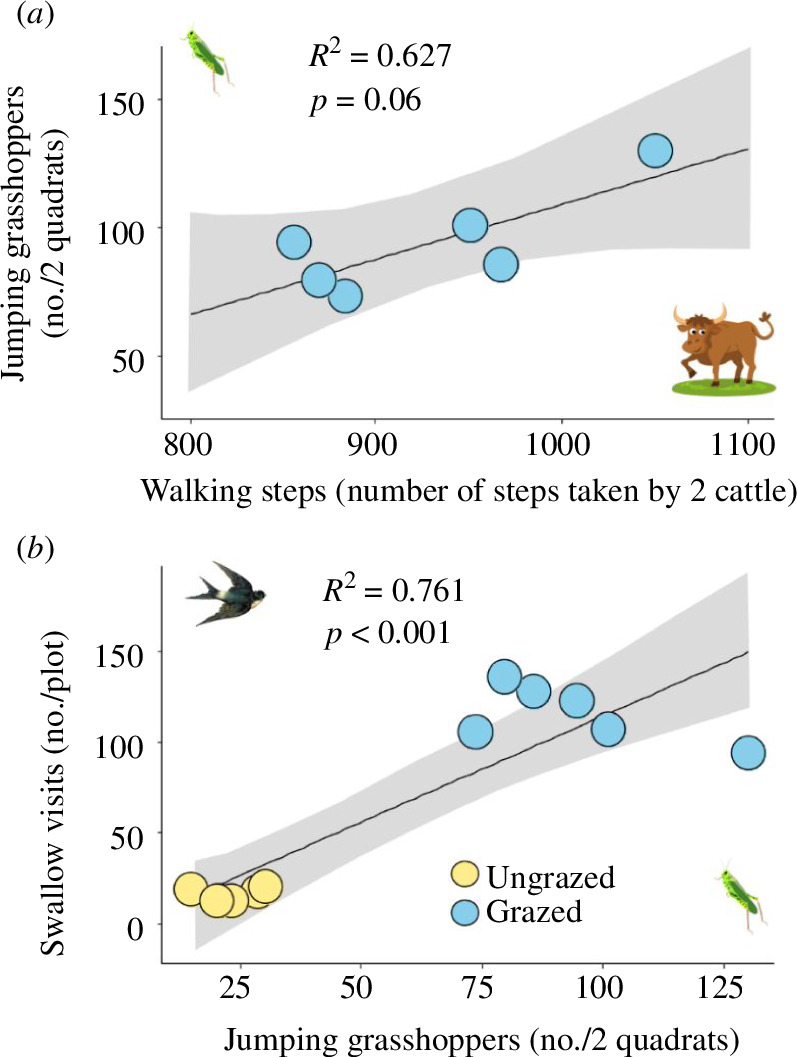
The relationships among cattle, grasshoppers and swallows in ungrazed and grazed plots in August of 2021. (*a*) The effects of the cattle walking step count (measured as number of steps taken by 2 cattle) on the number of jumping grasshoppers and (*b*) effects of the number of jumping grasshoppers on swallow visit frequency.

Compared with the ungrazed plots, cattle grazing significantly increased the number of jumping grasshoppers (*F*_1,10_ = 68.776, *p* < 0.001; figure 2*a*) and the rate of swallow visits to the grazed plots (*F*_1,5_ = 268.34, *p* < 0.001; figure 2*b*). Cattle grazing decreased grasshopper abundance from 13.4 individuals m^−2^ in ungrazed plots to 6.3 individuals m^−2^ in grazed plots (*F*_1,10_ = 17.393, *p* = 0.002; figure 2*c*). The number of jumping grasshoppers tended to be positively associated (nearly significant) with the number of cattle steps in grazed plots (*R* ^2^ = 0.627, *F*_1,4_ = 6.711, *p* = 0.06; figure 3*a*). We also found a positive association between the frequency of swallow visits and the number of jumping grasshoppers (*R* ^2^ = 0.761, *F*_1,10_ = 31.87, *p* < 0.001; figure 3*b*) across all grazed and ungrazed plots. Cattle grazing did not affect plant cover for any of the functional plant groups (i.e. *L. chinensis* grass, other grasses and forbs; electronic supplementary material, figure S3).

### (b) Behavioural restrictions on grasshopper jumping

**Figure 4.**
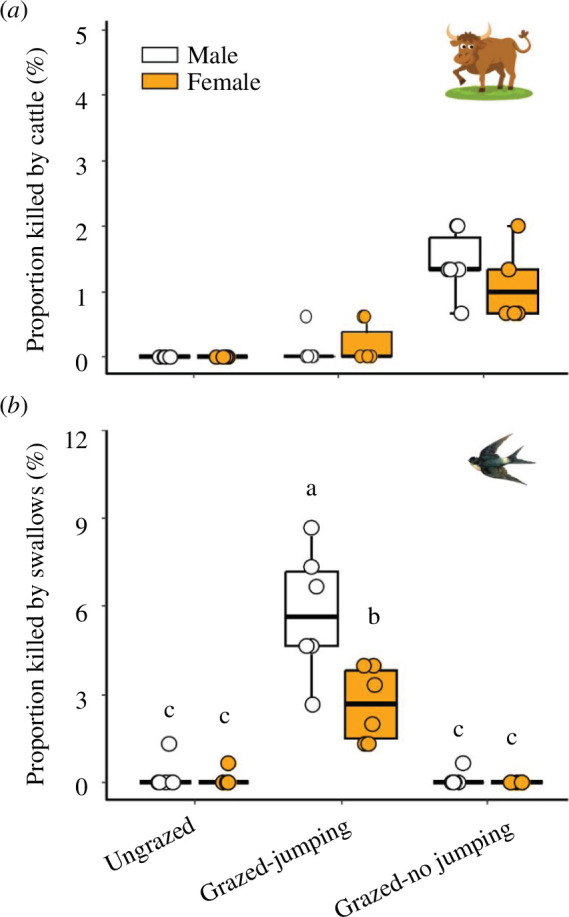
Effects of grasshopper jumping behaviour on their predation risk by cattle and swallows in ungrazed and grazed plots in August of 2022. The proportion of grasshoppers killed was evaluated in the three experimental treatments: grasshoppers that were able to jump in an ungrazed plot (ungrazed), grasshoppers that were able to jump in grazed plots (grazed–jumping) and grasshoppers that were unable to jump in grazed plots (grazed–no jumping). Different letters above the columns indicate significant differences among the three treatments using Tukey multiple comparisons (*p* < 0.05). Error bars represent ± s.e.

Grasshoppers’ jumping ability affects their vulnerability to incidental consumption or trampling by cattle (*χ* ^2^ = 11.320, *p* = 0.05; figure 4*a*). Grasshoppers able to jump in the grazed plots were almost never killed by cattle (i.e. 0–0.2% mortality rate from cattle). But in the grazed plots where grasshoppers were unable to jump, the number of males and females killed by cattle was 12-fold higher (i.e. 1.4% morality rate in the field) and fourfold higher (1.1% morality rate) comparted with those with jumping ability in the grazed plots (male: *z* = 2.482, *p* = 0.06; female: *z* = 2.085, *p* = 0.16; figure 4*a*).

When jumping was not prevented, cattle grazing increased the number of grasshoppers that were consumed by swallows 24-fold compared to those in the ungrazed plots (grazing: *χ* ^2^ = 30.90, *p* < 0.001; figure 4*b*). There was no interaction between grazing treatment and grasshopper sex (*χ* ^2^ = 0.008, *p* = 0.93), yet male grasshoppers experienced predation risk that was more than double that experienced by female grasshoppers (sex: *χ* ^2^ = 10.58, *p* = 0.001). The analysis used to assess the differences in the proportions of male and female grasshoppers killed by swallows among the three treatments indicated that shifts in jumping behaviours were the key determinant of swallows’ predation success of (*χ* ^2^ = 58.28, *p* < 0.001; figure 4*b*). Tukey *post-hoc* tests revealed that the mortality of both male and female grasshoppers that were not prevented from jumping was significantly greater than the mortality risk experienced by grasshoppers that were prevented from jumping in the grazed plots or by those that were able to jump in the ungrazed plots.

## 4. Discussion

We provided empirical evidence that LMH grazing can regulate food-web dynamics in grasslands via pathways that are not mediated by plants. Consistent with our hypotheses, we found that cattle grazing increased the jumping activity of grasshoppers, enabling them to avoid being incidentally consumed or trampled by cattle. The cattle-induced escape jumps, in turn, made grasshoppers more vulnerable to the risk of avian predation (figure 1). Overall, cattle grazing resulted in substantially lower grasshopper density in the grazed compared with the ungrazed fields.

In the absence of LMH, *Euchorthippus* grasshoppers spent more than 98% of their time within the vegetation canopy, and they rarely jumped [51]. Swallows hunt predominantly on the wing for air-borne arthropods and pose little risk to plant-dwelling insects [38,52]. However, approaching LMH induced grasshopper escape jumps, making them susceptible to swallow predation. Swallows responded to the grasshoppers’ behavioural change by foraging more in the grazed plots (figure 2). This suggests that swallows indirectly benefit from cattle grazing by getting access to a valuable food resource that was otherwise largely inaccessible for them. The cattle-induced increase in grasshopper jumping likely enhances the swallows’ ability to detect the grasshoppers, and allows them to intercept the grasshoppers in mid-air and away from risky obstacles like branches and fences [53]. As such, our study provides the first quantification of the underlying behavioural mechanism that likely govern the well-acknowledged LMH–bird interactions reported in many ecosystems [32–35]. Whether or not LMH grazing can be used as a cost-effective management tool to support insectivorous bird populations in grasslands worldwide deserves further attention (also see [54]).

LMH can affect smaller herbivores via plant-mediated interactions by altering the quantity and/or nutritional quality of plant resources [14,41,43]. Additionally, they can influence smaller herbivores by reducing the complexity of vegetation structure, which increases their vulnerability to predators [10,55]. Yet, LMH may also affect food-web dynamics by incidentally consuming or trampling plant-dwelling invertebrates. A DNA-metabarcoding analysis that examined how frequently LMH ingest arthropods revealed that more than 70% of cattle faeces contained plant-dwelling arthropods, but only rarely jumping insects like grasshoppers [25]. Moreover, molecular data indicate that in our study system cattle rarely consume grasshoppers (R Forman and M Inbar, unpublished data). Our behavioural manipulation coincided with this result, showing that even when the grasshoppers’ ability to jump was restricted, incidental consumption and trampling by cattle were responsible for only 1–2% of grasshopper mortality in the grazed plots (figure 4*a*). This result, along with other studies [24,25], indicates that direct intra-guild predation of large grazers on grasshoppers is seemingly small.

The cattle-induced escape jumps significantly increased the vulnerability of grasshoppers to swallow predation, as the number of grasshoppers consumed by swallows increased 37-fold when compared with grasshoppers that were prevented from jumping in the grazed plots (figure 4*b*). These findings raise the question of why grasshoppers jump and expose themselves to high risk of avian predation. We hypothesize that an approaching LMH poses an immediate and largely deterministic risk of mortality to the grasshopper. The grasshopper must jump to effectively mitigate this imminent risk. Thus, at the individual level, escaping from LMH is expected to be adaptive even at the cost of a potential increase in the risk of avian predation. The population consequences of these behaviours may depend on the type and density of LMH and those of the coexisting avian predators [14,56]. The population consequences of LMH grazing on arthropod herbivores may also depend on the arthropod’s species-specific foraging domain and escape behaviour. For instance, unlike grasshoppers, aphids and ladybirds drop off plants when approached by LMH [27,57]. These escape responses my increase the risk of starvation for arthropods and the risk of predation by small terrestrial predators, but not by avian predators.

Our findings highlight the important role that LMH may play in influencing trophic dynamics. We demonstrated the indirect, non-consumptive mechanism by which LMH can shape the inter-specific relationships and community structure in grasslands. Escaping from LMH may expose arthropod herbivores to danger from predators that pose little risk otherwise. Such changes in the strength of trophic interactions may cascade to affect other species that are not directly affected by LMH activity, substantially perturbing the entire food-web structure [9]. For example, lower grasshopper density owing to swallow predation may adversely affect populations of typical grasshopper predators and parasites, and also indirectly affect their alternative prey species and their predators [58]. Other ground predators such as amphibians and reptiles, which may also have found the escaping grasshoppers to be easy prey, are likely to face similar risks to the grasshoppers: being forced to move and being exposed to avian predators [30]. Thus, the indirect interactions induced by LMH are likely to be more important, common and complex than was previously thought [3,24]. Future work should quantify those cascading effects while controlling for direct consequences of LMH grazing on the focal species, for instance by preventing plant biomass removal by LMH or by excluding avian predators from the experimental grazing plots.

Cattle grazing was associated with substantial reduction in grasshopper density. This dramatic decline may be attributed in part to the higher risk of swallow predation (figure 3). Bird predation has been shown to control the density of grasshoppers in various grazed systems. For example, bird predation explained a 58% decline in grasshopper (*Dissosteira carolina*, *Melanoplus bivittatus*, *Arphia pseudonietana*) densities in prairie [59] and a 25% decline in all grasshopper abundance in the sandhills grassland of North America [60]. Yet, the observed decline in grasshopper density cannot be attributed solely to cattle-induced changes in grasshopper behaviour. Cattle grazing can reduce the vegetation’s structural complexity, exposing plant-dwelling arthropods to increased avian predation. Moreover, Zhu *et al*. [41] found that cattle grazing in our study system can exert a negative effect on *Euchorthippus* grasshopper fitness and population dynamics by increasing the leaf nitrogen (N) content of *L. chinensis* grasses, the grasshoppers’ preferred food plant. The relative contributions of these forces in shaping grasshopper population dynamics are difficult to distinguish in the field. Regardless, our work clearly demonstrates that large herbivores can exert indirect effects on arthropods that are not mediated by changes in the properties of plant communities.

We found sex differences in grasshopper vulnerability to swallow predation that coincide with previous findings from the North American prairies (figure 4*b*). There, male grasshoppers were twice as susceptible to bird predation than females [39], likely because male grasshoppers jump and fly more frequently than females. Additionally, males may be more susceptible because they expose themselves more while searching for females [61]. The observed sex-specific differences in grasshopper vulnerability to swallow predation may affect the sex ratio and potentially the demography of the grasshopper population. This may expose yet another mechanistic pathway by which LMH grazing indirectly influences arthropod populations and the community as a whole.

In conclusion, we found that grasshoppers evade approaching cattle by jumping out of the vegetation. This escape behaviour rendered them more vulnerable to swallow predation and negatively affected their population size. Typically, LMH are expected to affect food-web dynamics indirectly by altering the composition and structure of plant communities (plant-mediated pathways). We instead provided evidence that LMH can also shape food-web dynamics by altering the behaviour of plant-dwelling herbivores (behaviour-mediated pathways) and that this prevalent interaction can control the population size of common arthropod herbivores via increased avian predation. Our investigation was on domestic cattle and we focused on the mechanistic details of how grasshoppers respond to an approaching LMH, which is likely applicable to both domesticated and wild LMH of similar sizes. It is well known the avian predators such as cattle egrets also follow grazing wild ungulates in natural habitats (e.g. [62]). As we have shown here, the behaviour-mediated effects induced by LMH unravel a largely convoluted pathway by which anthropogenic activities may impact species interactions and ecosystem processes. Globally, LMH assemblages are undergoing rapid transformations [5]. Human activities lead to dramatic population declines in wild mammalian herbivores but at the same time to a significant increase in livestock populations. Introduction of LMH into novel grazing areas, or dramatic changes in grazing intensities in existing rangelands, are likely to play a pivotal role in shaping species interactions and the community structure of grasslands in our rapidly changing world. These changes may have profound downstream effects on the provision of various ecosystem services in grasslands worldwide.

## Data Availability

Data are available from the Dryad Digital Repository [63]. Supplementary material is available online [64].
